# Bilateral Primary Adrenal Non-Hodgkin Lymphoma

**DOI:** 10.4274/tjh.2013.0195

**Published:** 2014-06-10

**Authors:** Vehbi Erçolak, Oğuz Kara, Meral Günaldı, Çiğdem Usul Afşar, Berna Bozkurt Duman, Arbil Açıkalın, Melek Ergin, Şeyda Erdoğan

**Affiliations:** 1 Harran University Faculty of Medicine, Department of Medical Oncology, Şanlıurfa, Turkey; 2 Çukurova University Faculty of Medicine, Department of Medical Oncology Adana, Turkey; 3 Adana Training and Research Hospital, Department of Medical Oncology, Adana, Turkey; 4 Çukurova University Faculty of Medicine, Department of Pathology, Adana, Turkey

**Keywords:** non-Hodgkin lymphoma, Lymphoid cells neoplasms, B-Cell neoplasms

## TO THE EDITOR

Non-Hodgkin lymphoma (NHL) is found in the adrenal gland secondarily at a rate of 25% [1]. Primary adrenal lymphoma (PAL) is found in fewer than 1% of NHL cases [2]. Secondary adrenal gland involvement is usually unilateral, while PALs are usually bilateral [3,4]. Primary adrenal gland lymphomas are usually diffuse large B-cell lymphomas (DLBCL) [1,5]. Most cases are of B-cell origin [4]. 

**Figure 1:**
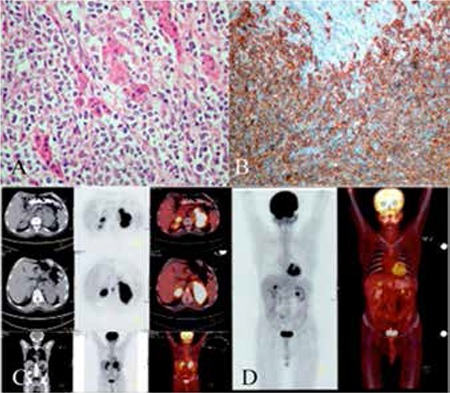
Atypical lymphoid cells of middle-large diameter with oval-round, prominent vesiculated nuclei and diffusely invading adrenal tissue (A), lymphoid cells were diffusely CD20-positive and normal adrenocortical cells were found smashed in between them (B), before treatment (C), after 4 cycles of chemotherapy (D).

A 62-year-old male admitted to our hospital with abdominal pain in the left lumbar region persisting for 4 months without B-symptoms. Abdominal and thorax computed tomography (CT) scanning was performed and revealed a mass of 93x60 mm on the left adrenal gland and a 58-mm mass on the right adrenal gland. The mass was nonfunctional according to hormone test results. The patient underwent left adrenalectomy. The pathology specimen revealed NHL, DLBCL, leukocyte common antigen (+), CD20 (+), CD3 (-) (Figures-1A,1B). In positron emission tomography (PET)-CT, there was an advanced level of hypermetabolic mass with metastatic lymphadenopathy in the left mesenteric region and retrocrural regions (Figure-1C). There was no malignancy in PET-CT after performing 4 cycles of R-CHOP (rituximab, cyclophosphamide, doxorubicin, vincristine, and prednisone) chemotherapy (Figure-1D). Chemotherapy was completed in 6 doses, and 5 months after chemotherapy, the patient had no signs of clinical, laboratory, or radiological progression. Written informed consent was optained from the patient.

PAL are rare, generally occurring among patients of advanced age (mean: 68 years) and dominantly in males (M/F: 2.2/1) [1,6]. Cases are majorly found bilaterally (approximately 70%) [1,7]. Bulky disease is more common [4,7]. Clinical symptoms of PAL include local symptoms, systemic symptoms compatible with adrenal insufficiency [4,5]. Adrenocortical insufficiency is observed in 50% of patients and there is no correlation with tumor size [1,2]. Adrenocortical insufficiency occurs when there is more than 90% destruction in the adrenal parenchyma [5].

Nonspecific clinical presentation and imaging results make it very hard to diagnose before surgery [5]. In CT and MRI, PAL is seen as a complex mass with variable density [1]. The diagnosis of PAL is confirmed only with pathological evaluation [5]. 

Prognosis is usually poor. Poor prognostic factors are advanced age, large tumor size, bilateral involvement, high LDH levels, involvement of other organs, and adrenal insufficiency at admission [1,3,5,7,8]. Nongerminal B-cell phenotype and Bcl-6 rearrangement is associated with poor prognosis, as represented in the literature [2]. Patients with 3 or more risk factors (international prognostic index (IPI) scores) are accepted poor prognoses [4].

Treatment includes surgery, combination chemotherapy and radiotherapy, but bilateral adrenalectomy with adjuvant radiotherapy is still controversial [7]. Commonly used chemotherapy regimens are CHOP [8]. Response rates are relatively low and permanent remission is rare. In a review of 83 patients, the 1-year survival rate was 17.5% [4]. Full or partial treatment response is seen in only 1/3 of cases [9]. Surgical resection when used alone is related to poor prognosis in tumors with aggressive histopathological subtypes. Radiotherapy is usually not a part of treatment in the beginning, but it could be used in low-grade lymphomas and incomplete surgical excision or after chemotherapy with positive functional tumors in radiographic imaging in residual disease [4].

In conclusion, this rare disease should be kept in mind in patients with adrenal masses even in the absence of other malignancies, without nodal or extranodal involvement, or in patients with adrenal insufficiency.

## CONFLICT OF INTEREST STATEMENT

The authors of this paper have no conflicts of interest, including specific financial interests, relationships, and/ or affiliations relevant to the subject matter or materials included. 

## References

[1] Wang J, Sun NC, Renslo R, Chuang CC, Tabbarah HJ, Barajas L, French SW (1998). Clinically silent primary adrenal lymphoma: a case report and review of the literature. Am J Hematol.

[2] Mozos A, Ye H, Chuang WY, Chu JS, Huang WT, Chen HK, Hsu YH, Bacon CM, Du MQ, Campo E, Chuang SS (2009). Most primary adrenal lymphomas are diffuse large B-cell lymphomas with non-germinal center B-cell phenotype, BCL6 gene rearrangement and poor prognosis. Mod Pathol.

[3] Hsu CW, Ho CL, Sheu WH, Harn HJ, Chao TY (1999). Adrenal insufficiency caused by primary aggressive NHL lymphoma of bilateral adrenal glands: report of a case and literature review. Ann Hematol.

[4] Grigg AP, Connors JM (2003). Primary adrenal lymphoma. Clin Lymphoma.

[5] Ezer A, Parlakgümüş A, Kocer NE, Çolakoğlu T, Nursal GN, Yıldırım S (2011). Primary adrenal non-Hodgkin’s lymphoma: report of two cases. Turk J Gastroenterol.

[6] Kumar R, Xiu Y, Mavi A, El-Haddad G, Zhuang H, Alavi A (2005). FDG-PET imaging in primary bilateral adrenal lymphoma: a case report and review of the literature. Clin Nucl Med.

[7] Aziz SA, Laway BA, Rangreze I, Lone MI, Ahmad SN (2011). Primary adrenal lymphoma: differential involvement with varying adrenal function. Indian J Endocrinol Metab.

[8] Kim KM, Yoon DH, Lee SG, Lim SN, Sug LJ, Huh J, Suh C (2009). A case of primary adrenal diffuse large B-cell lymphoma achieving complete remission with rituximab-CHOP chemotherapy. J Korean Med Sci.

[9] Yang Y, Li Q, Pan Y (2010). Bilateral primary adrenal lymphoma. Br J Haematol.

